# Cognizant Fiber-Reinforced Polymer Composites Incorporating Seamlessly Integrated Sensing and Computing Circuitry

**DOI:** 10.3390/polym15224401

**Published:** 2023-11-14

**Authors:** Mohammed Jaradat, Jorge Loredo Duran, Daniel Heras Murcia, Leah Buechley, Yu-Lin Shen, Christos Christodoulou, Mahmoud Reda Taha

**Affiliations:** 1 Department of Civil and Infrastructure Engineering, Al-Zaytoonah University of Jordan, Amman 11733, Jordan; m.jaradat@zuj.edu.jo; 2 Department of Computer Science, University of New Mexico, Albuquerque, NM 87131, USA; jloredo98@unm.edu (J.L.D.); buechley@unm.edu (L.B.); 3 Gerlad May Department of Civil, Construction & Environmental Engineering, University of New Mexico, Albuquerque, NM 87131, USA; dherasmurcia@unm.edu; 4 Department of Mechanical Engineering, University of New Mexico, Albuquerque, NM 87131, USA; shenyl@unm.edu; 5 Department of Electrical and Computer Engineering, University of New Mexico, Albuquerque, NM 87131, USA; christos@unm.edu

**Keywords:** fiber-reinforced polymers, self-sensing, smart composites, piezoresistive sensors, cognizant composites, resilient infrastructure

## Abstract

Structural fiber-reinforced polymer (FRP) composite materials consisting of a polymer matrix reinforced with layers of high-strength fibers are used in numerous applications, including but not limited to spacecraft, vehicles, buildings, and bridges. Researchers in the past few decades have suggested the necessary integration of sensors (e.g., fiber optic sensors) in polymer composites to enable health monitoring of composites’ performance over their service lives. This work introduces an innovative cognizant composite that can self-sense, compute, and implement decisions based on sensed values. It is a critical step towards smart, resilient infrastructure. We describe a method to fabricate textile sensors with flexible circuitry and a microcontroller within the polymer composite, enabling computational operations to take place in the composite without impacting its integrity. A microstructural investigation of the sensors showed that the amount of oxidative agent and soaking time of the fabric play a major role in the adsorption of polypyrrole (PPy) on fiberglass (FG). XPS results showed that the 10 g ferric chloride solution with 6 h of soaking time had the highest degree of protonation (28%) and, therefore, higher adsorption of PPy on FG. A strain range of 30% was achieved by examining different circuitry and sensor designs for their resistance and strain resolution under mechanical loading. A microcontroller was added to the circuit and then embedded within a composite material. This composite system was tested under flexural loading to demonstrate its self-sensing, computing, and actuation capabilities. The resulting cognizant composite demonstrated the ability to read resistance values and measure strain using the embedded microcontroller and autonomously actuate an LED light when the strain exceeds a predefined limit of 2000 µε. The application of the proposed FRP system would provide in situ monitoring of structural composite components with autonomous response capabilities, as well as reduce manufacturing, production, and maintenance costs.

## 1. Introduction

Fiber-reinforced polymer (FRP) composites are widely used worldwide in numerous applications, including the automotive, aerospace, and construction industries. FRPs are materials used across various fields due to their unique properties and advantages. Carbon fiber-reinforced polymer (CFRP) stands out for its exceptional mechanical properties, excellent fatigue resistance, corrosion resistance, and high load-bearing capacity [1,2]. Other FRPs, like basalt fiber-reinforced polymer (BFRP) and glass fiber-reinforced polymer (GFRP) [3], offer good mechanical properties and are readily available from abundant sources. However, their long-term performance does not match the durability of CFRP. Despite this limitation, they find application in industries where the superior properties of CFRP are not essential. Overall, FRPs are valued for their remarkable mechanical attributes and resistance to various forms of degradation, making them vital materials in structural engineering applications. This work utilizes a vinyl ester/glass fiber composite material which is extensively used in industrial and marine applications, and its reliability and durability have been thoroughly studied [4,5,6,7,8,9,10,11].

Obtaining in situ information regarding FRP composite parts throughout their life cycle is essential to enabling functional composites [12,13,14]. The advances in manufacturing methods and computational algorithms, including machine learning, have led researchers to intensify their efforts to produce smart devices and materials [15]. Advanced composite materials are typically defined as autonomous, with capabilities such as sensing a particular stimulus or actuating in response to that stimulus [16,17,18,19,20].

Integrating sensing into FRP composites has been part of the work on structural health monitoring (SHM) research for the past three decades. Most of the previous work focused on using some type of sensor to monitor a specific feature. This approach is limited to one sensory function only in FRP composites and can be categorized into two broad categories: external sensing and self-sensing. The former utilizes off-the-shelf sensors that can be surface-mounted. A “MEMS” accelerometer has been used to track crack growth in a composite structure by monitoring the drift in the material compliance [21]. Piezoelectric transducers have been utilized to detect features such as loading/unloading and boundary condition changes through the generation of lamb waves, which are analyzed using signal processing techniques [22]. Sensors can also be embedded within the composite. Fiber optic strain sensors were used to monitor pultruded FRP bars and composite marine structures [6,8]. Piezoelectric sensors were embedded within FRP composites and used for the characterization of the material and damage monitoring [23,24]. Yang et al. utilized capacitive sensors and light-emitting diodes (LEDs) to produce a multifunctional composite seven-segment display capable of displaying 128 symbols [25]. However, the application of off-the-shelf sensors suffers from several drawbacks, such as their inability to comply with the geometry changes of the deformed composites and the difficulty of embedding them in specific locations. They can also have a disruptive impact on the performance of the composite [26].

On the other hand, self-sensing means that the composite material becomes a sensor, thereby avoiding issues related to sensor integration in the composite. The use of conductive fabrics within the composite to serve as sensors is a common approach used in many studies. For instance, inherently conductive fabrics such as carbon fibers can act as strain sensors in a composite by exploiting electrical resistance changes due to certain fiber failure mechanisms [27,28]. Nonconductive fabrics, however, need to be transformed into conductive ones. Several methods were used, such as using carbon nanotubes (CNTs) growth on glass fibers [29,30,31,32] or directly coating the fibers with carbon nanomaterials [33]. In situ polymerization of pyrrole with fabrics in the reaction medium is a promising approach that results in electrically conductive polypyrrole (PPy)-coated fabrics. Intrinsically conducting polymers (ICP) such as PPy have been used in fabricating strain sensors by acting as fillers in a polymer or fibrous substrate [34,35,36,37,38,39]. Furthermore, it has been used on different fabrics, such as silk [40], polyester [41], cotton [42], and wool fibers [43]. However, no studies were performed on using PPy-coated FG strain sensors. Additionally, most of the applications of PPy-coated fabric sensors were focused on highly stretchable materials with very high failure strains. Whereas in this work, the PPy-coated FG strain sensors are used to sense structural composites that exhibit relatively low failure strains. It is worth mentioning that the adsorption of PPy to fabrics is a complex phenomenon due to its dependency on various fabric parameters [43].

The concept of actuation in response to a stimulus is ubiquitous in nature, such as pinecones opening and closing in response to humidity to release seeds or sunflowers following the sun to maximize photosynthesis [44]. Inspired by nature, many composite material systems were developed to respond to various stimuli such as heat, stress, and electrical current, among others [45]. Nevertheless, a truly smart material system must be adaptive by having the ability to sense, compute (i.e., perform a series of feature extraction and pattern recognition processes [46] as critical components of the SHM paradigm), and make decisions based on the former computation to enable a specific response [47]. A computing capability connected to the sensing and actuating capabilities is necessary to develop cognizant FRP composites. To avoid integration issues due to the rigidity of conventional printed circuit boards (PCBs), fabric PCBs can be used. The use of a flexible circuit allows for attaining multiple sensory abilities at once. Therefore, the material will have a more advanced method of self-monitoring. Flexible PCBs are used extensively in electronic devices to interconnect different components together in medical devices, cameras, cell phones, etc. [48]. They usually utilize a plastic substrate, such as polyester, polyimide, or poly(vinylidene fluoride-co-trifluoroethylene) (PVDF), to create flexible PCB via photolithography and other processes [49,50,51]. Lv et al. [52] utilized flexible PCBs to attach sensor matrices for in situ composite production monitoring. Jang et al. [53] used flexible circuitry to develop a multifunctional spacecraft sandwich composite panel that possesses thermal, radiation shielding, and electrical functions in addition to its load-bearing abilities. However, in the present study, an electronic textile approach was followed [48]. The fabrication method applied herein allows for seamless and local integration between the electronic components and the fiber-reinforced structural composite material. In this work, a further step is taken towards creating a cognizant material that can compute and logic the data received from its sensory functions and has the ability to induce actions.

**Figure 1:**
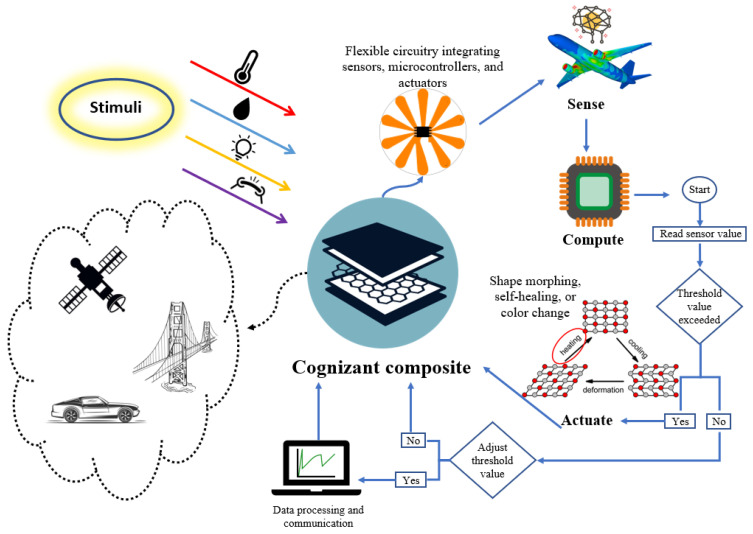
Conceptual schematic of a cognizant FRP composite material system.

This work introduces a “*cognizant FRP composite*” that can self-sense, compute, and implement decisions based on sensed values, as described in Figure 1. Figure 1 illustrates the capacity of a cognizant FRP composite structure to autonomously perceive various stimuli, including temperature, moisture, light, and strain alterations, among other factors. Leveraging its inherent self-sensing abilities, the cognizant FRP composite possesses the capability to interpret and measure these stimuli, subsequently making informed decisions guided by predetermined threshold values and/or pre-coded algorithms. Consequently, the cognizant FRP composite exhibits the potential to engage in responsive actions facilitated by mechanisms like shape morphing, self-healing, and color transformation, among other dynamic processes.

**Figure 2:**
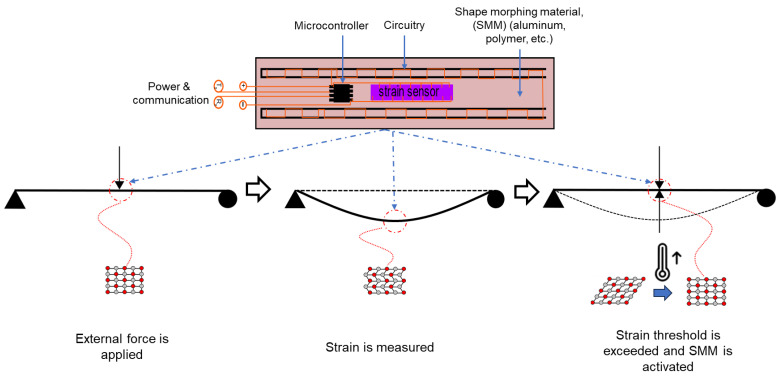
Schematic representation of the proposed cognizant FRP composite.

Conceptually, piezoresistive woven fiberglass (FG) can be developed through in situ polymerization of pyrrole onto FG. This conductive fabric undergoes changes in resistance as it is mechanically strained. Therefore, this fabric can monitor strain by correlating resistance change values with strain values, and a strain sensor can be created for the self-sensing composite. The sensor can then be embedded in an FRP composite made from the same woven fiberglass. Flexible circuitry is constructed from a conductive fabric. The circuitry is then used to build an array of electronics, including computing circuitry to provide power, enable communication to the sensor, and compute based on the sensed values. The resulting material is a cognizant FRP composite that can be tested under stress to demonstrate the system’s self-sensing, self-computing, and response capabilities. The response can be heating the shape-morphing material to induce a counter curvature in the FRP composite. Alternatively, turning on a light indicator connected to the computing circuitry when the strain exceeds a pre-coded threshold can also demonstrate the computing capability of the new cognizant FRP composite. The proposed cognizant FRP composite is depicted in Figure 2.

## 2. Experimental Methods

### 2.1. Materials, Design, and Fabrication

Plain weave fiberglass reinforcement was used for its ease of handling, good resin penetration, and air removal during fabrication. The woven fiberglass used is an E-glass fabric with an average weight of 123 g/m^2^ and a fabric thickness of 0.15 mm supplied by US Composites (Palm Beach, FL, USA). The vinyl ester Hydrex^®^ 100 33350, provided by US Composites (Palm Beach, FL, USA), was used as the resin system. It is pre-promoted to cure at room temperature with methyl ethyl ketone peroxide (MEKP) as a catalyst. The mixing ratio of the catalyst to the resin used was 1.25% of the resin weight. This resin has a relatively low viscosity of 500 cps, which helps in the fiber impregnation during fabrication, and a gel time of 35–40 min. The tensile strength, modulus, and strain to failure are 83 MPa, 3.5 GPa, and 4%, respectively. Pyrrole (98% pure) acquired from Fischer Scientific (Waltham, MA, USA) was used as the monomer to prepare the fiberglass sensor. Ferric chloride hexahydrate, also from Fisher Scientific, was used as the oxidizing agent.

The piezoresistive sensors were produced following the technique described by Honnet et al. [54] for piezoresistive textiles. This technique utilizes an in situ polymerization process of the pyrrole monomers using ferric chloride as an oxidant. First, a fiberglass sheet was cut and stitched on the edges to prevent fraying during polymerization. A solution of 1 L of water and 25 mL of pyrrole was prepared. The fiberglass sheet was then soaked in the solution and stirred. The polymerization process starts once an aqueous ferric chloride solution is added to the mixture. As the polymerization process progresses, the solution and the fiberglass begin to turn to a black color. The amount of ferric chloride and the fiberglass sheet soaking time varied, as depicted in Table 1. Finally, the fiberglass is removed from the mixture, rinsed, and air-dried. The required fiberglass (FG) sensor was cut from the fiberglass sheet using a Glowforge 3D laser cutter (Seattle, WA, USA).

**Figure 3:**
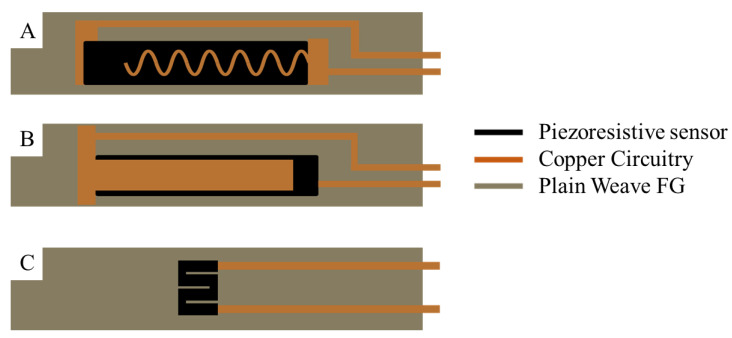
Sensor designs (**A**) copper-wavy (**B**) copper-solid (**C**) No copper.

To be able to utilize the piezoresistive sensors, circuitry constructed from conductive fabric was added. The conductive fabric used is a copper-plated polyester taffeta from LessEMF (Latham, NY, USA) with a thickness of 0.08 mm, a weight of 80 g/m^2^, and a surface resistivity of 0.05 Ohms/square. Three types of sensor designs were prepared for this work. The first type, shown in Figure 3A, has a wavy pattern of copper fabric spreading across the FG sensor. This design is motivated by a desire to maintain sensor sensitivity. The ideal sensor includes a stable electrical and mechanical connection between the conductive fabric and a large sample of the polymerized material’s surface. In contrast, the second design (Figure 3B) employs a solid piece of copper fabric over the polymerized material. In this design, we lose sensor sensitivity but are assured of a robust connection between the two materials. Sensors A and B are designed to detect compression and tension. The electrical resistance of the polymerized material changes as it is compressed. The placement of copper electrodes on the top and bottom of the polymerized material allows us to sense changes through the *z*-axis of the material, i.e., changes that occur during a three-point bend test.

The third sensor, Figure 3C, has copper fabric connected to it at two terminal locations. This configuration allows us to sense resistance changes across the XY length of the polymerized material, i.e., changes during a tensile strength test. The different sensor configurations enable measuring changes in resistance that correlate to strain changes.

**Figure 4:**
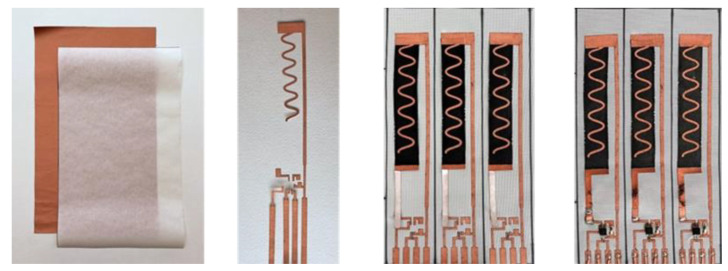
Sensor and circuit construction process. The figure shows an example of the construction of a wavy sensor design with the capacity to integrate a microcontroller and LED light. From left to right: Fiberglass and copper fabric sheets, the copper is laser-cut and then attached to the fiberglass sheet and strain sensor, and finally components are soldered to the circuit.

The circuitry construction was carried out following the techniques described by Buechley and Eisenberg [48]. Figure 4 shows the construction stages of a design. The circuit was cut in the shape shown in Figure 4 using a laser cutter. The sensor configuration was then adhered to the fiberglass through the heat-activated adhesive on the back of the conductive fabric.

**Figure 5:**
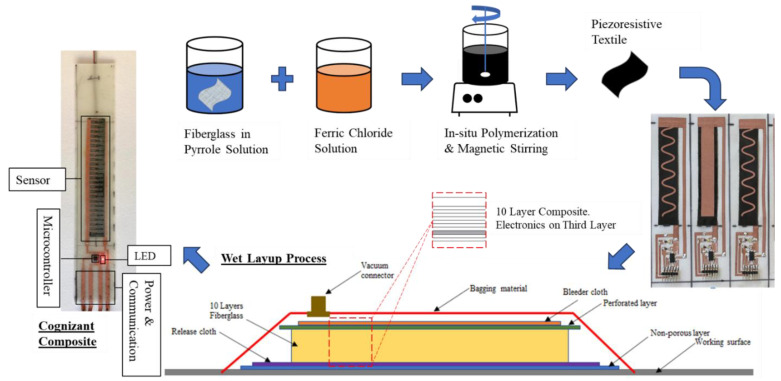
Synthesis of FG sensors and incorporation into a composite material using the wet layup process.

All the specimens used in this work were cut from vinyl ester glass fiber composite plates fabricated using a vacuum-assisted hand layup process. Each plate consists of 10 glass fiber sheets. The third sheet of the composite was prepared with the circuitry, an ATTINY85 microcontroller, and an LED light soldered to the circuit. To reduce stress concentrations and the possibility of defects during the fabrication process due to the inclusion of the microcontroller, the FG sheets on top of the microcontroller were cut using the laser cutter to produce a hole that would fit the protruding microcontroller. This fabrication method provides a smooth finish without any wrinkling or compromise to the specimen’s integrity. Furthermore, the microcontroller was placed in a low-stress location within the composite material. It is located at the ends of the specimen to avoid the area of maximum stress at the mid-span during a bending test. Each specimen has four wires extending out of the composite material. The specimens with microcontrollers had sockets soldered to the circuit to allow for power and communication with the microcontroller. All composite plates were left under vacuum at room temperature for 24 h at a vacuum pressure of 2.3 × 10^−2^ Torr. The plates were then placed in the oven for 12 h at 80 °C for post-curing. The specimens were cut out of the plates using a waterjet cutter to produce specimens for mechanical testing. Figure 5 shows the overall process of building the polymerized FG sensors, constructing the sensors, and fabricating the composite using a wet layup process.

### 2.2. Surface Analysis

Hitachi S-5200 Nano Scanning electron microscopy (SEM) was employed to observe the surface morphology of the sensors using an acceleration voltage of 2 kV. X-ray photoelectron spectroscopy (XPS) measurements were carried out using a Kratos AXIS Ultra photoelectron spectrometer from Kratos Analytical (Manchester, UK). The XPS system has a monochromatic Al anode and a non-monochromatic Mg x-ray source. All measurements taken were an average of 3 different areas for each sample.

### 2.3. Thermogravimetric Analysis

Thermogravimetric analysis (TGA) was carried out using a TA Instruments Discovery TGA 55 device. The temperature was increased from 50 to 800 °C at a heating rate of 10 °C/min under an air atmosphere. Uncoated fiberglass and coated fiberglass with different concentrations of iron chloride and different soaking times in the polymerization medium were compared. The thermal stability of specimens was examined, and the PPy content was determined.

### 2.4. Mechanical Testing

**Figure 6:**
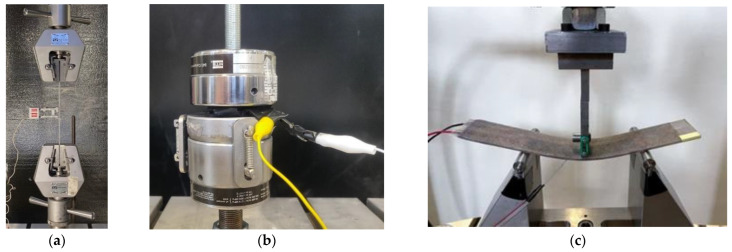
(**a**) Uniaxial tension test (**b**) Pressure test (**c**) Three-point bending test.

A series of mechanical tests, shown in Figure 6, were conducted to evaluate the FG piezoresistive sensor’s mechanical and electrical resistance performance before and after embedment into a composite material system. The evaluation is performed by testing vinyl ester GFRP composite with FG sensors using three-point bending and uniaxial tension tests. The universal testing machine (UTM) used for both tests is a Bionix servohydraulic system that has a load cell capacity of 25 kN (±1 N) and a maximum stroke of 130 mm (±1 mm). All tests were conducted in a displacement-controlled mode at a 2 mm/min constant rate. The sampling rates of the flexural and tensile tests were 1024 Hz and 1 Hz, respectively, and the data was collected through the FlexStar MTS^®^ 793 data acquisition system. The FG sensor data was collected using an Arduino Uno microcontroller.

The uniaxial tension test was performed to evaluate the structural integrity of the GFRP composite. The test was conducted following ASTM D3039. The specimen dimensions are 250 mm long, 21 mm wide, and 2.7 mm thick, with a span length of 170 mm. The strain was measured using an axial extensometer attached to the specimen with a gauge length of 25.4 mm and a strain range of +50% and −20%, which is sufficient for FRP composites.

A uniaxial compression test was carried out on the FG sensor before embedment into a resin matrix as part of a structural composite material. The FG sensors were sandwiched between two rigid copper electrodes and then compressed until full contact was accomplished, and the FG sensor resistance values were recorded.

Finally, a three-point bending test was conducted to evaluate the resistance performance of the FG piezoresistive sensors in a composite material. The piezoresistive sensors were placed on the third layer from the bottom of the 10-layer composite to capture the strain of the outermost fibers. The tested samples’ dimensions are 170 mm × 36.7 mm × 2.4 mm. The test was performed following ASTM D790. The span length of the tested samples is 100 mm. The strain was recorded using pre-wired, linear pattern strain gages supplied by Omega Engineering (Norwalk, CT, USA). The strain gauges were attached at the midspan of the surface, closer to where the piezoresistive sensor is located. The specimens were placed in the testing setup so that the sensors were on the bottom surface of the composite for tensile strain measurement.

## 3. Computing Circuitry Integration and Testing

**Figure 7:**
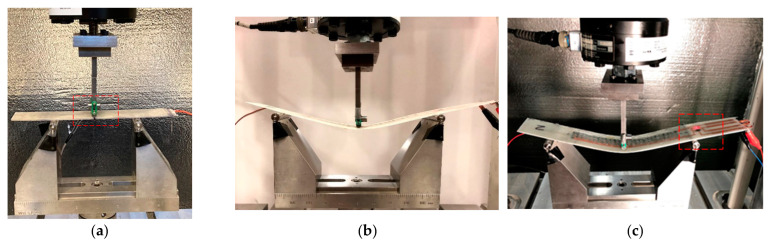
Integrated computing circuitry. (**a**) An external force is applied. (**b**) Strain is measured and computed. (**c**) The strain threshold is exceeded, and the LED light (red square) is activated.

We also constructed a simple prototype that included an integrated microcontroller and an LED light as an example of an actuator to represent the capability of the integrated computing circuitry, as shown in Figure 7. In this simple proof-of-concept prototype, the composite was subjected to bending stress produced by a three-point loading test according to ASTM D790. The microcontroller in the embedded and integrated computing circuitry was programmed to switch on the LED light when the strain values exceeded a predetermined threshold of 2000 µε.

## 4. Results and Discussion

### 4.1. X-ray Photoelectron Spectroscopy (XPS) Analysis

**Figure 8:**
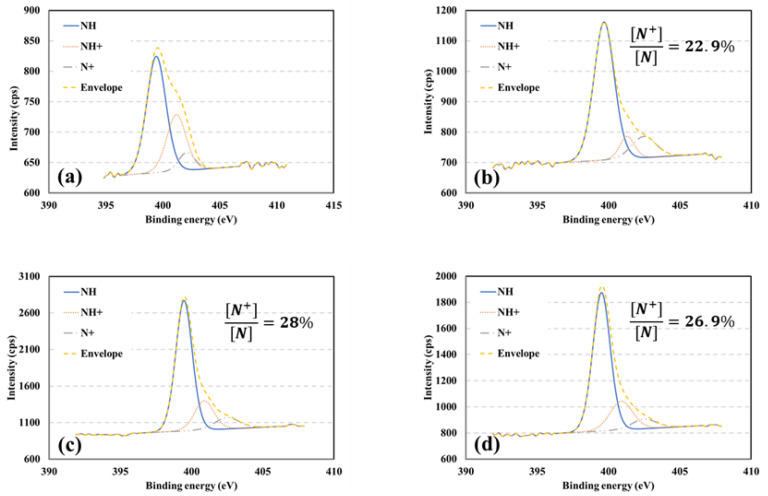
XPS high-resolution scans of FG sensors (**a**) Neat (**b**) FG Sensor 1 (**c**) FG Sensor 2 (**d**) FG Sensor 3.

High-resolution scans of N1s obtained through XPS and describing the in situ polymerization of fiberglass are shown in Figure 6. The graphs are fitted with three decomposed Gaussian peaks at 399.6, 401.5, and 402.5 eV, corresponding to benzenoid amine (-NH-), protonated benzenoid amine (-N+H-), and protonated quinonoid imine (-N+=), respectively. XPS is commonly utilized to investigate protonation and doping levels of PPy as an indication of electrical conductivity by determining the ratio of positively charged nitrogen N+ to the total nitrogen content N_total_ [52,55,56]. This ratio, representing the degree of protonation, was obtained by integrating the fitted peaks. The values of the ratios are presented in Figure 8. The FG Sensor 2 displayed the highest degree of protonation, followed by the FG Sensor 3, and finally the FG Sensor 1. It can be observed that the soaking time significantly impacts the adsorption of PPy on the fiberglass during the in situ polymerization process. In addition, increasing the amount of the oxidative agent in the polymer does not lead to a higher degree of protonation. Interestingly, Lv et al. [52] found that increasing the amount of pyrrole to ferric chloride improves the conductivity of the soaked fabric or textile.

### 4.2. Scanning Electron Microscope Analysis (SEM)

**Figure 9:**
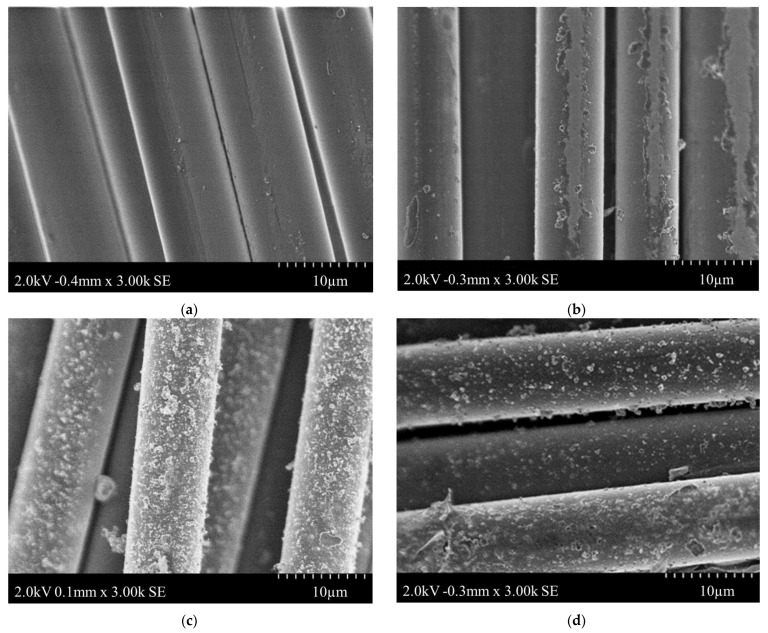
SEM images (**a**) Neat FG (**b**) FG Sensor 1 (**c**) FG Sensor 2 (**d**) FG Sensor 3.

The morphology of the FG sensors was studied using SEM to examine the PPy depositions on the fabric. SEM images of neat fiberglass and PPy-coated fiberglass are depicted in Figure 9. No depositions are observed on the neat fiberglass. Whereas for the PPy-coated fiberglass, more PPy is deposited on the surface of the FG Sensor 2 when compared with the FG Sensor 3 and the FG Sensor 3. PPy particles are distributed in a way that creates a uniform coating on the surface of the fiberglass without creating clusters of PPy.

### 4.3. Thermal Analysis

**Figure 10:**
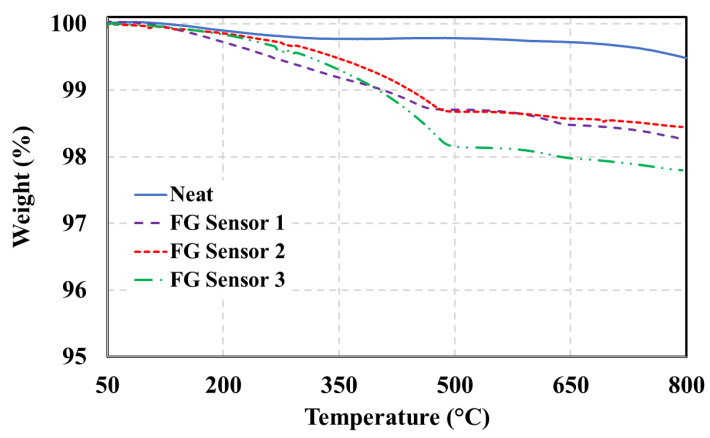
TGA of FG sensors.

The thermal degradation of coated specimens can be divided into three regions, as depicted in Figure 10. The first region extends up to 145 °C and represents losses due to moisture evaporation and volatile impurities. The second stage starts around 200 °C, reported by [42,43] as the onset of PPy degradation, and is sustained until 490 °C–500 °C. Finally, at 500 °C and up until 800 °C, these minor losses are mainly due to the degradation of sizing, coating, or primer treatment applied to fiberglass during manufacturing. This is supported by the fact that the degradation between 500 °C and 800 °C is about 0.3% in all tested specimens. Notably, fiberglass does not degrade below 1000 °C due to its inorganic nature. Furthermore, upon the completion of the TGA test, the tested sensors showed a white color instead of the initial black color of the FG fabric, indicating the absence of all deposited PPy on the FG surface. Thus, temperatures higher than 800 °C would not have been useful in evaluating the thermal degradation of PPy deposited on the FG surface. The thermal stability of the sensors was evaluated by calculating the gradient of the weight loss curves in Figure 10. A steep slope indicates a low thermally stable fabric. It can be noticed that a higher soaking time creates a more thermally stable fabric, as evidenced by the lower slope of degradation of the FG Sensor 2.

### 4.4. Mechanical Analysis

**Figure 11:**
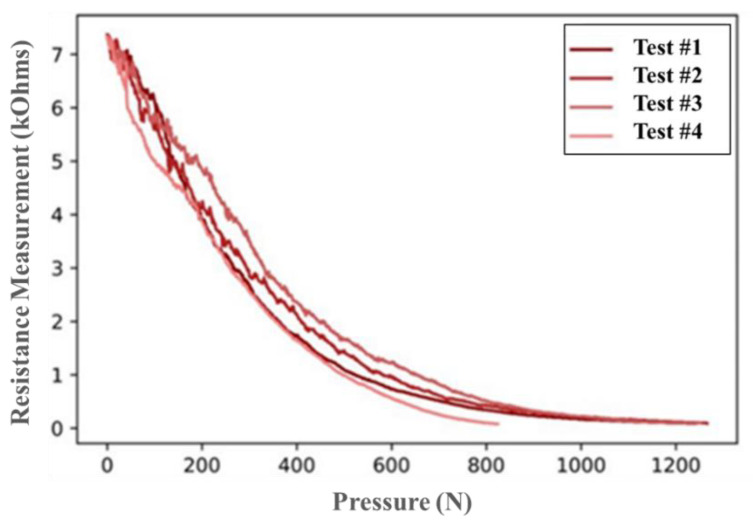
Pressure test of the FG sensor.

Pressure test results on the FG sensors are shown in Figure 11. The results are consistent among the tested samples and show that the resistance drops exponentially as the FG sensor gets compressed. This is due to the gradual increase in contact between the conducting PPy deposited on FG yarns as the pressure increases. The SEM images presented previously show the PPy deposited on the yarns, and it can be observed that with compression, the distance between PPy deposits will keep getting smaller. This allows the material to become further conductive through an increase in the number of conductive paths, and consequently, the resistance drops. These results align with the findings presented in the literature [57,58]. Aly K et al. [57] tested piezoresistive CNTs sheets functioning as strain sensors and embedded them into woven fiberglass composites. Luo et al. [58] monitored the fabrication of a composite plate using a vacuum-assisted wet layup method, which puts the composite material under compression. Both works observed that the resistance of the piezoresistive sensor drops in a nonlinear exponential manner due to compression.

**Figure 12:**
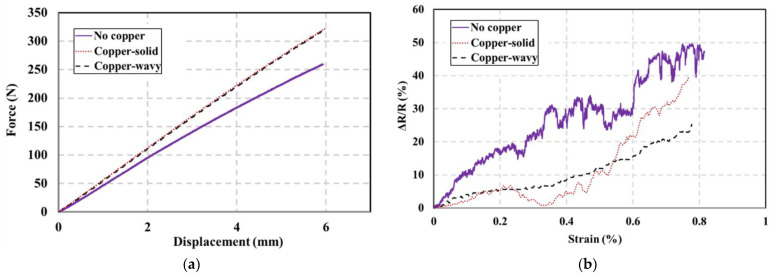
Results of the three sensor configurations tested in a three-point bending (**a**) force-displacement (**b**) change in resistance vs. strain.

Figure 12a shows the force-displacement response of the composite samples undergoing bending and containing different sensor configurations. The T-test showed no significant difference between the copper-solid and copper-wavy. However, they significantly differ from the no-copper configuration, which contains less FG due to the smaller piezoresistive sensor used. Figure 12b shows the change in resistance recorded from the FG sensors versus strain values obtained from strain gauges in a three-point bending test. All the designs showed an increase in resistance as the strain increased, which indicates that tensile strains are dominant in this test. Strains resulting from tensile stresses cause the PPy particles to pull apart from each other, leading to a reduction in conductive pathways and, consequently, an increase in resistance. This increase in resistance was observed by Sebastian et al. while tensile testing CNTs-coated fiberglass operating as the sensor in a fiberglass composite material [29]. The copper-wavy FG sensor outperformed the solid-copper sensor configuration due to smaller surface area contact between the circuit and piezoresistive FG sensor, producing a consistent response with no sudden drops in its behavior. The copper-solid configuration has the largest surface area contact between the copper and FG sensor. A large contact area increases the possibility of delamination between the copper and the sensor and, therefore, jeopardizes the integrity of the circuit.

Furthermore, the no-copper configuration measures the resistance changes between two discrete points at the terminals of the sensor fabric located directly under the point of load application. Therefore, the resistance readings experienced some variability when compared to the other two configurations, as depicted in Figure 12b. Overall, the copper-wavy sensor configuration performed better than the other two configurations, as it exhibited less variability and more consistency in its response. Despite the fluctuation in resistance change, the copper-wavey FG sensor showed a consistent increase in resistance with the increase in strain, proving its ability to work as a sensor for detecting increased internal strains in the FRP composite.

**Figure 13:**
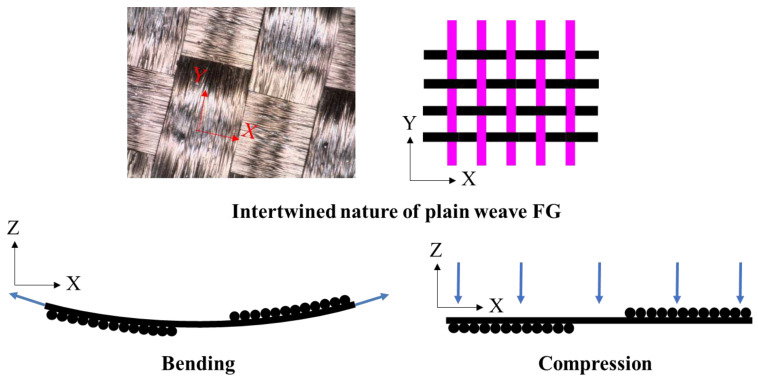
Schematic representation showing the different effects of bending (resulting in tension stresses represented by the in-plane arrows) and compression stresses (represented by the out-of-plane arrows) of the FG sensor and its significance on sensor reading variability.

Pressure tests on the polymerized FG sensors showed good consistency within the tested samples as opposed to the bending test results, where higher variability exists. Figure 13 shows schematically how the two tests affect the yarns of the FG in different ways. The FG sensor in compression records the out-of-plane strains it is experiencing under a uniform pressure exerted on the specimen as a whole, which leads to a consistent change in resistance, whereas in bending, the FG sensor experiences a combination of in-plane and out-of-plane strains (tension and compression) due to a pressure that is exerted on the specimen in a localized manner. Therefore, variability in resistance changes in bending is higher than in compression, especially at the midspan, where the load is applied directly to the specimen.

This combination of strains affects the change in resistance of the FG sensors, leading to high variability among test samples. Moreover, the mode of interaction between the intertwining yarns of the FG sensor affects the resistance readings.

**Figure 14:**
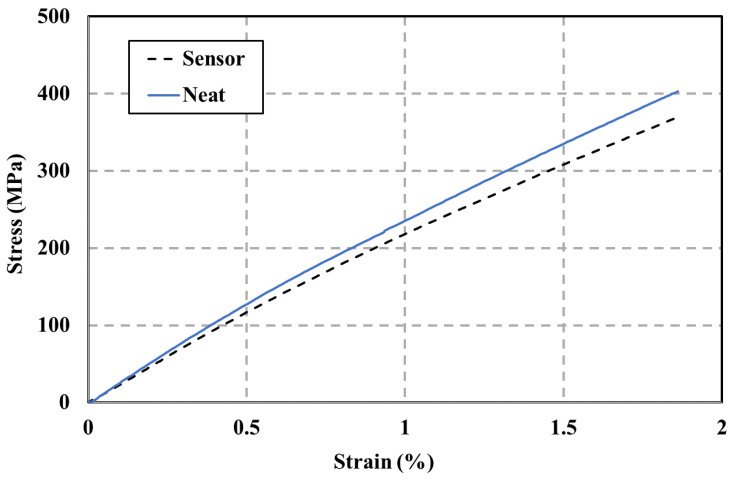
Structural integrity tensile test results show the stress-strain of the neat GFRP sample and the GFRP sample incorporating the wavy FG sensor. The graph indicates that the FG wavy sensor can be embedded in the GFRP composites and has no effect on the structural integrity of the GFRP composite (the curves shown are median curves from 5 test specimens).

Figure 14 shows stress versus strain plots of the tension test of the neat GFRP sample (no sensor) versus the GFRP sample incorporating the wavy FG sensor. As shown in Figure 14, no significant difference in strength or stress-strain behavior between the two composites can be observed. A two-tailed T-test was performed on the stress and strain data for both composites with and without the FG sensors to evaluate the impact of embedding the FG sensors on the integrity of the GFRP composite. The results of the stress test are P (T ≤ t) 0.56 and P (T ≤ t) 0.88 for strain. Therefore, issues related to the formation of defects or locations of high-stress concentrations are eliminated, and the FG sensors can be seamlessly embedded into the GFRP composites without impacting their structural integrity.

**Figure 15:**
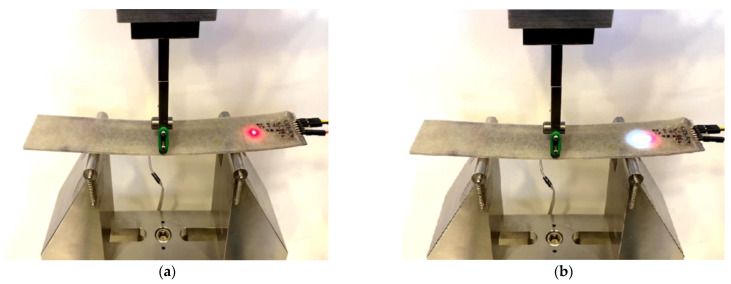
Microcontroller integrated into FRP composite material with LED indicator. (**a**) LED is in standby mode as bending of the sample progresses (**b**) Strain exceeding a threshold of 2000 µε, and LED light turns on.

The embedded microcontroller was programmed to switch on the LED light when the strain exceeded 2000 µε. The strain-resistance relationship shown in Figure 11 (copper-wavy) was used to monitor the strain through the change in resistance of the piezoresistive PPy-FG sensor. An increase in the change in resistance by 5% indicates exceeding the set strain threshold of 2000 µε. The LED light was in standby mode, as shown in Figure 15a, as long as that threshold was not exceeded. As the bending increases, the change in resistance also increases. Due to the variability that exists in sensor readings, the LED light starts blinking as the threshold is exceeded at times. Once the microcontroller starts reading consistent values showing an increase in resistance of 5%, the LED light remains on, as depicted in Figure 15b.

The present study successfully demonstrates the feasibility of seamlessly integrating self-sensing and computing elements into FRP composites. Nonetheless, integrating microcontrollers into FRP composites introduces a significant concern about preserving structural integrity. We are currently working on strategies to minimize this impact by selecting the location of such controllers away from high-stress regions. Further research is warranted to study the reinforcement of the composite parts proximal to the microcontroller so that any loss of mechanical properties can be minimized, facilitating an upscale of the technology. Furthermore, the reliability of the produced sensors should be further examined by testing the composite material under various environmental conditions, such as temperature and humidity, as well as assessing the fatigue strength under cyclic loading. We believe that the ability to integrate sensors, circuitry, and computing elements paves the road to cognizant FRP composites. However, further research is needed to reduce variability and noise in the FG sensor’s performance.

Cost analysis for producing cognizant FRP composite materials involves key material, design, and fabrication cost considerations. In terms of design considerations, it is essential to carefully evaluate sensor placement, considering the specific data requirements and desired responses for particular structural elements. While the fiberglass used in making the sensors is a relatively minor cost factor, additional expenses are incurred through the procurement of the polymer (pyrrole) and the oxidizing agent (ferric chloride) for sensor production. Among these materials, ferric chloride is relatively inexpensive compared with pyrrole, which has a relatively high cost. However, both pyrrole and ferric chloride are utilized in relatively small quantities, with pyrrole at 25 mL per liter of solution and ferric chloride at 10–20 g per liter of solution. Consequently, we anticipate that the cost of the materials will have only a negligible impact on the overall cost of the composite. On the other hand, the fabrication process, which includes sensor soaking, polymerization, and laser cutting, represents a notable cost and time investment. Finally, the integration of sensors and circuitry, once produced, into the composite is seamless and does not significantly add to fabrication cost or time. Furthermore, low-cost microcontrollers proved effective and improved the overall affordability of the sensor system. It is therefore apparent that sensor fabrication might be the component with the highest cost implications in the production of the cognizant composite. Scaling up production would entail replicating the sensor production process for each part, potentially leveraging automation while integrating sensors into the composite fabrication. This approach might potentially represent an economical means of manufacturing cognizant composites with self-sensing and computing capabilities.

Furthermore, further research shall also account for embedding actuating materials such as SMAs. SMAs are known for their ability to undergo reversible shape changes in response to temperature fluctuations. SMAs can thus counteract the curvature effects and balance the FRP composite stress once the strains exceed a predetermined threshold. Additionally, we envision incorporating AI algorithms into the computing circuitry to perform feature extraction and pattern recognition. Building upon earlier work utilizing fuzzy damage pattern recognition [59], we aspire to leverage AI’s capabilities to identify and cognize intricate damage patterns within the composite structure of the FRP composite and make a selection of a myriad of actions through a series of embedded SMAs to recover this damage and extend the life of the FRP composite. Establishing efficient point-to-point connections for local communication is crucial in contemplating structural scale-up, requiring precise sensor design and placement [60]. From a computational perspective, the challenge lies in coordinating local interactions to monitor global behavior [60].

## 5. Conclusions

In this study, we fabricated and tested a new type of FRP composite material that integrates sensing, computing, and response capabilities. Several sensory structures were constructed using a piezoresistive sensor attached to a stretchable circuit that combines a microcontroller as a computing chip and an LED as a response indicator.

The sensor was created by coating FG fabric with conductive PolyPyrrole through an in situ polymerization process to seamlessly integrate it into the FG composite material. The process parameters of this polymerization process were varied and studied using XPS, SEM, and TGA. The XPS analysis revealed that soaking time and oxidative agent amount influenced the protonation and doping levels of PPy. The SEM images showed that more PPy was deposited on FG with higher degrees of protonation. The XPS analysis revealed that a 10 g ferric chloride solution with a soaking time of 6 h achieved the highest degree of protonation (28%). The images also showed a uniform distribution of PPy particles on the FG surface without creating clusters. TGA demonstrated that a longer soaking time resulted in a thermally stable PPy-coated FG fabric. The sensors were then mechanically characterized using pressure tests. The tests showed the repeatability of their behavior and that their resistance decreased exponentially as they were compressed.

After the sensor was embedded during the fabrication of the structural FRP composite, a mechanical assessment of the composite was conducted through flexural and tensile tests to evaluate both the sensor’s performance and the composite’s structural integrity resulting from the sensor integration. Flexural tests showed that the performance of sensory structures with surface contact between circuits and piezoresistive FG exhibits less variability. Among the sensor designs we explored, the wavy pattern exhibited the least resistance variability, with a range of 30%. This finding highlights the importance of sensor design in achieving consistent and reliable measurements. Nevertheless, this contact’s surface area determines the sensor’s consistency, which is affected by possible delamination issues.

The tensile tests confirmed that the sensors could be embedded seamlessly into the composite without impacting the FRP’s structural integrity. Tests also showed successful integration of computing circuitry and controllers in the FRP composite, with an LED indicator showing light flashes as the FRP strain exceeded a predetermined threshold. The cognizant composite demonstrated precise strain monitoring, triggering an LED light when strain exceeded 2000 µε, corresponding to a change in resistance of 5%.

This study demonstrates the feasibility of incorporating sensing, circuitry, and computing components into structural FRP composites without compromising their structural integrity. This work paves the way for developing “*cognizant FRP composites*” that can sense and respond to structural health information quickly and locally. The proposed cognizant FRP composites can potentially eliminate or reduce the conditions resulting in inducing damage in the FRP by integrating AI algorithms programmed on the computing circuitry embedded in the FRP composites to perform damage pattern recognition and take actions “e.g., inducing counter rotations” to eliminate or reduce the potential damage. The integration of PPy-coated woven FG as a piezoresistive strain sensor, the innovative fabrication method inspired by electronic textiles, and the local integration of a microcontroller within the composite material represent key novel aspects of the study. This holistic approach results in a seamless blend of structural and computational elements, paving the way for the development of cognizant FRP composites necessary for future resilient infrastructure. Further research is warranted to include actuation systems (e.g., SMA) that can produce this counteraction to extend the service life of FRP composites. Additional work is warranted to reduce the variability in performance and develop calibration procedures. Further work is also needed to examine the cost implications and feasibility of large-scale production of the proposed cognizant FRP composites. While our research primarily focuses on the innovative aspects of these composites and their potential advantages, we recognize that a thorough exploration of economic and practical considerations is essential to evaluating their scale-up applicability comprehensively.

## Figures and Tables

**Table 1:** Polymerization time and ferric chloride amounts used in FG sensors.

FG Sensor	Ferric Chloride (g)	Soak Time (h)
FG Sensor 1	10	1
FG Sensor 2	10	6
FG Sensor 3	20	6

## Data Availability

Available upon request.
